# Supplementary material for: Spatial and Bioaccumulation of Heavy Metals in a Sheep-Based Food System: Implications for Human Health
Source: Toxics. 2024 Oct 16;12(10):752. doi: 10.3390/toxics12100752 (PMC11511467; doi:10.3390/toxics12100752)

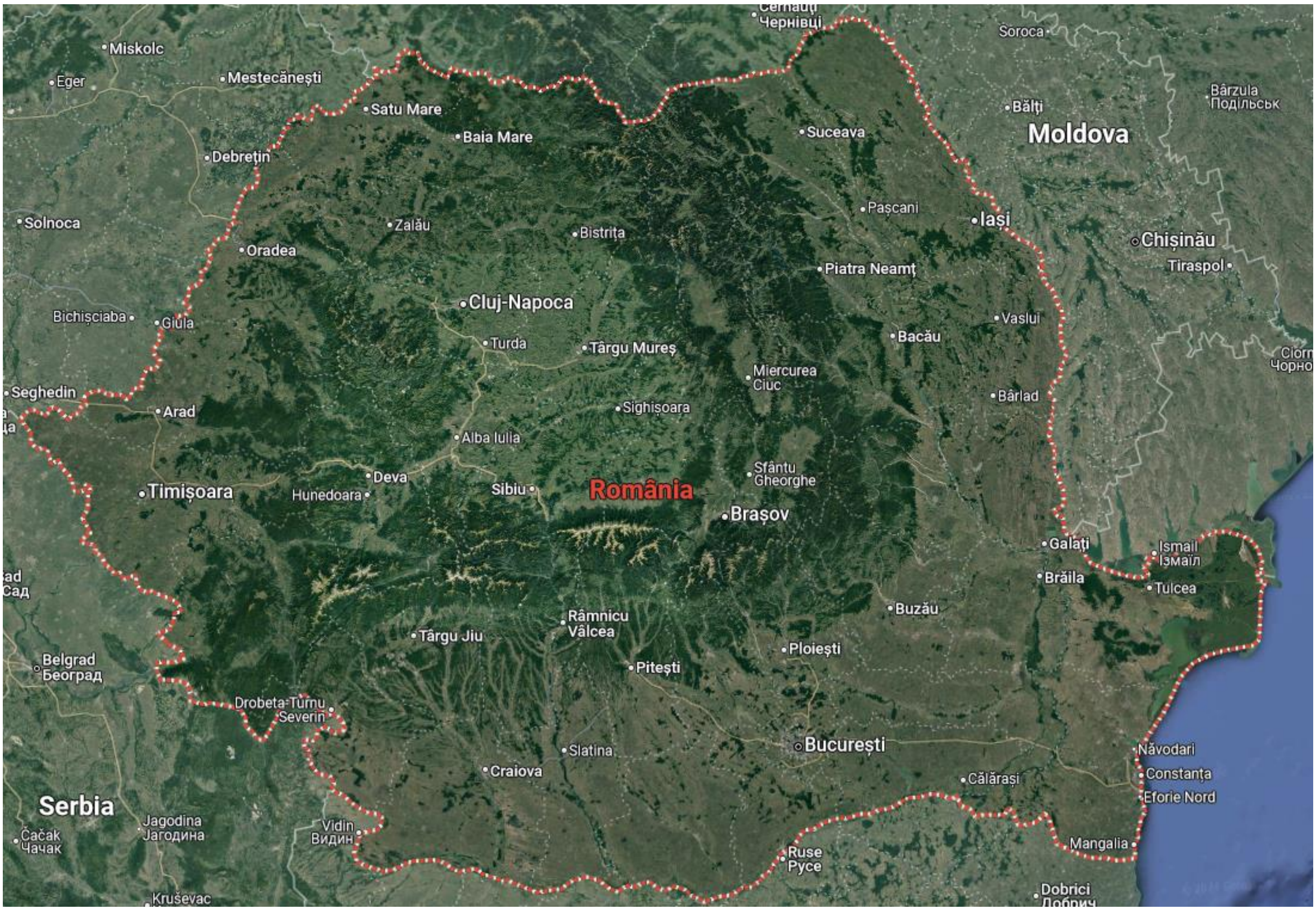

The control zone is the North-Western region of Romania, encompassed by the cities of Maramureș (Baia Mare) and Bistrița-Năsăud (Tîrlișua)

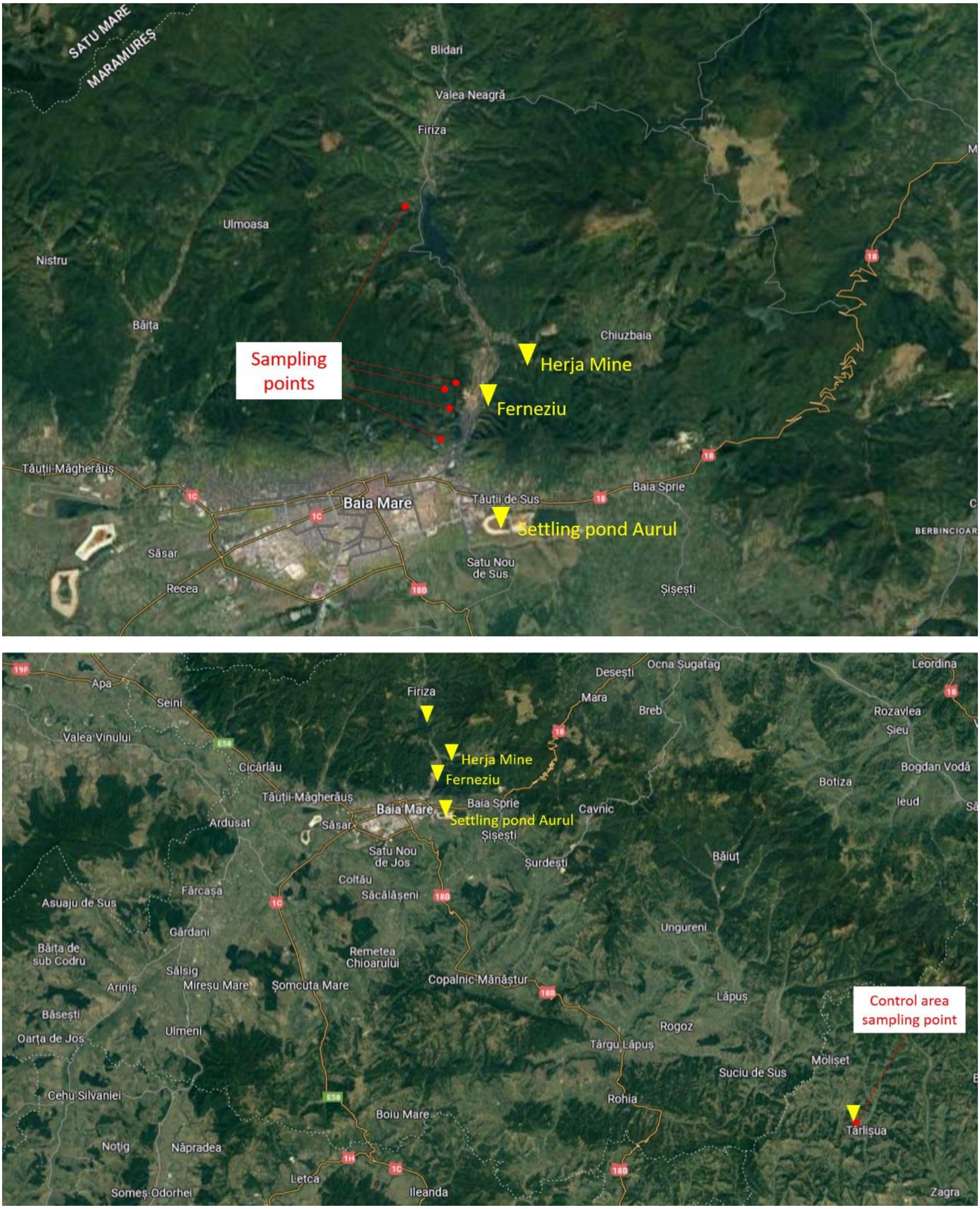

Figure S1. Geographical origins of soil, green grass, milk, cheese, and sheep serum samples. Sample codes denote both sample type and associated pollution source.

Table S1. Soil sampling location in Ferneziu, Firiza (Maramureș area) and Tîrlișua (Bistrița-Năsăud), Romania.

Sample code	Number of samples	Harvest period	Soil details	Sampling depth (cm)	Geographical origin	Distance from the source of pollution (~) km	Area/ Country	Year of harvest	Environment	Anthropogenic influence
Soil samples exposed to anthropogenic sources of heavy metals pollution										
S_{1-2024} S_{2-2024} S_{3-2024} S_{4-2024} S_{5-2024} S_{6-2024} S_{7-2024} S_{8-2024} S_{9-2024} $S_{10-2024}$ $S_{11-2024}$ $S_{12-2024}$	A set of three sub-soil samples was collected from each sampling point	08-10 May	The sample collection location coincides with semi-permanent grazing ground utilized ty sheepfold	0 – 10 cm	Ferneziu	8.0 km to the Herja mine / 11.5 km to the Herja mine	Maramureș Country/ Romania	2024	Rural	Near (~ 10/12 km distance) to the Herja Mine
$S_{13-2024}$ $S_{14-2024}$ $S_{15-2024}$ $S_{16-2024}$ $S_{17-2024}$ $S_{18-2024}$ $S_{19-2024}$ $S_{20-2024}$ $S_{21-2024}$ $S_{22-2024}$ $S_{23-2024}$ $S_{24-2024}$	A set of three sub-soil samples was collected from each sampling point	08-10 May	The sample collection location coincides with semi-permanent grazing ground utilized ty sheepfold	0 – 10 cm	Ferneziu	5.5 km to the Herja mine / 7.5 km to the Herja mine	Maramureș Country/ Romania	2024	Rural	Near (~ 6/7 km distance) to the Herja Mine
$S_{25-2024}$ $S_{26-2024}$ $S_{27-2024}$ $S_{28-2024}$	A set of three sub-soil samples was	20-24 May	The sample collection location coincides	0 – 10 cm	Firiza	16.5 km to settling	Maramureș Country/	2024	Rural	Near (~ 17 km distance) to settling pond mining (decant pond) Aurul from Tăuții de Sus

S29-2024<br>S30-2024<br>S31-2024<br>S32-2024<br>S33-2024<br>S34-2024<br>S35-2024<br>S36-2024	collected from each sampling point		with semi-permanent grazing ground utilized ty sheepfold			pond Aurul<br>17.0 km to settling pond Aurul				
Background soil samples										
S37-2024<br>S38-2024<br>S39-2024<br>S40-2024<br>S41-2024<br>S42-2024<br>S43-2024<br>S44-2024<br>S45-2024<br>S46-2024<br>S47-2024<br>S48-2024	A set of three subsoil samples was collected from each sampling point	06-07 June	The sample collection location coincides with semi-permanent grazing ground utilized ty sheepfold	0-10 cm	Tîrlișua	The control area was specifically chosen due to the absence of both detectable heavy metal contamination and high traffic volume.	Bistrița-Năsăud Country/ Romania	2024	Rural	The control area was meticulously selected to ensure minimal to no potential influence from heavy metal contamination from the outset.

The linear distance between the sheepfold and the polluted area was determined using a handheld GPS unit. Semi-permanent sheep shelters offer a compelling combination of durability, relocatability, and cost-effectiveness, rendering them a versatile solution for contemporary sheep farming practices. This inherent adaptability to accommodate a wide range of flock sizes and specific requirements further underscores the practical value of semi-permanent sheep shelters.

Table S2. Detailed characterization of grassland vegetation and species composition within the study area

Agrostis capillaris (syn. *A. tenuis*) meadows dominate the vast expanses of hilly and lower mountainous regions, extending from an elevation of 300 meters to over 1200 meters [31]. Their presence spans the subzone of oaks and hornbeams to the understory of beech forests and beech-coniferous mixtures [31]. *Agrostis capillaris* meadows exhibit a diverse flora, encompassing species with high forage value alongside others with no nutritional worth or even detrimental or toxic properties [31]. Additionally, these meadows can be susceptible to encroachment by woody invaders such as hawthorn (*Crataegus monogyna*) and blackthorn (*Prunus spinosa*) in drier areas, while wetter zones may experience invasion by rosehip (Rosa canina), hazel (*Corylus avellana*), hornbeam (*Carpinus betulus*), and birch (*Betula pendula*) [31]. Despite potential management challenges related to unwanted vegetation, *A. capillaris* meadows can offer good pastoral value [31]. High-productivity meadows can reach a production of 10-15 t/ha dry matter (DM) and support a grazing capacity of 1.0-1.2 large ruminant units (LSU)/ha [31]. A second category of these meadows exhibits medium productivity, with a pastoral value of 5.0-7.5 t/ha DM and a grazing capacity of 0.5-0.8 LSU/ha [31].
Festuca rubra meadows are characteristic of the *Picea abies* (Norway spruce) forest floor, also known as the boreal zone [31]. Their altitudinal range extends from 700-800 m in some situations up to 1800 m in the Southern and Western Carpathians and 1600 m in the north of the Eastern Carpathians [31]. At lower elevations, these meadows intergrade with *Agrostis capillaris* communities. Overgrazing and declining soil fertility can lead to encroachment by low-value species like *Nardus stricta* (Moor grass) and *Deschampsia caespitosa* (Tufted hairgrass) [31]. Additionally, forbs with low forage value such as *Urtica sp.* (Nettles), *Veratrum album* (White hellebore), *Rumex sp.* (sorrels), and *Colchicum autumnale* (Autumn crocus) may become established [31]. The pastoral value of Festuca rubra meadows varies considerably, ranging from mediocre to good (5-15 t/ha dry matter and 0.5-1.5 large ruminant units/ha grazing capacity) [31].
Poa pratensis meadows, dominated by Kentucky bluegrass, are characteristic of the submontane zone (foothill region) in southern Romania, particularly within the distribution range of oak and hornbeam forests (sky and garniță forests) at low elevations (100-300 meters) on flat or gently sloping lands [31]. *Poa pratensis* is a mesophytic grass species with high forage value and palatability for grazing animals [31]. However, these meadows can also harbor weedy species with no nutritional value (e.g., bearded brome, sorrels, and meadow saffron) and even harmful or toxic plants (e.g., bittercress, thistles, and meadow crowfoot) [31]. Despite the presence of unwanted vegetation, Poa pratensis meadows offer good pastoral value, with a production potential of 7.5-12.5 t/ha dry matter (DM) and a grazing capacity of 1.0-1.5 large ruminant units (LSU)/ha [31].

Table S3. Sampling site of green grass from Ferneziu, Firiza (Maramureș area) and Tîrlișua (Bistrița-Năsăud), Romania.

Sample code	Number of samples	Harvest period	Green Grass details	Geographical origin	Distance from the source of pollution (~) km	Area/ Country	Year of harvest	Environment	Anthropogenic influence
Green grass samples exposed to anthropogenic sources of heavy metals pollution									
G1-2024<br>G2-2024<br>G3-2024<br>G4-2024<br>G5-2024<br>G6-2024<br>G7-2024<br>G8-2024<br>G9-2024<br>G10-2024<br>G11-2024<br>G12-2024	A set of three grass samples were collected from each sampling point	08-10 May	The sample collection location coincides with semi-permanent grazing ground utilized ty sheepfold	Ferneziu	8.0 km to the Herja mine<br>11.5 km to the Herja mine	Maramureș Country/ Romania	2024	Rural	Near (~ 10/12 km distance) to the Herja Mine
G13-2024<br>G14-2024<br>G15-2024<br>G16-2024<br>G17-2024<br>G18-2024<br>G19-2024<br>G20-2024<br>G21-2024<br>G22-2024<br>G23-2024<br>G24-2024	A set of three grass samples were collected from each sampling point	08-10 May	The sample collection location coincides with semi-permanent grazing ground utilized ty sheepfold	Ferneziu	5.5 km to the Herja mine<br>7.5 km to the Herja mine	Maramureș Country/ Romania	2024	Rural	Near (~ 6/7 km distance) to the Herja Mine
G25-2024<br>G26-2024<br>G27-2024<br>G28-2024	A set of three grass samples were	20-24 May	The sample collection location coincides	Firiza	16.5 km to settling	Maramureș Country/	2024	Rural	Near (~ 17 km distance) to settling pond mining (decant pond) Aurul from Tăuții de Sus

G29-2024<br>G30-2024<br>G31-2024<br>G32-2024	collected from each sampling point		with semi-permanent grazing ground utilized ty sheepfold		pond Aurul				
G33-2024<br>G34-2024<br>G35-2024<br>G36-2024					17.0 km to settling pond Aurul				
Background green grass samples									
G37-2024<br>G38-2024<br>G39-2024<br>G40-2024<br>G41-2024<br>G42-2024<br>G43-2024<br>G44-2024<br>G45-2024<br>G46-2024<br>G47-2024<br>G48-2024	A set of three grass samples were collected from each sampling point	06-07 June	The sample collection location coincides with semi-permanent grazing ground utilized ty sheepfold	Tîrlișua	The control area was specifically chosen due to the absence of both detectable heavy metal contamination and high traffic volume.	Bistrița-Năsăud Country/ Romania	2024	Rural	The control area was meticulously selected to ensure minimal to no potential influence from heavy metal contamination from the outset.

Table S4. Sampling site of sheep's milks and cheese from Ferneziu, Firiza (Maramureș area) and Tîrlișua (Bistrița-Năsăud), Romania.

Sample code	Number of samples	Harvest period	Milks and cheese details	Geographical origin	Distance from the source of pollution (~) km	Area/ Country	Year of harvest	Environment	Anthropogenic influence
Sheep's milks and cheese samples exposed to anthropogenic sources of heavy metals pollution									
M_{1-2024}	A set of three grass samples were collected from each sampling point	08-10 May	The sample collection location coincides with semi-permanent grazing ground utilized ty sheepfold	Ferneziu	8.0 km to the Herja mine	Maramureș Country/ Romania	2024	Rural	Near (~ 10/12 km distance) to the Herja Mine
C_{1-2024}									
M_{2-2024}									
C_{2-2024}									
M_{3-2024}									
C_{3-2024}									
M_{4-2024}									
C_{4-2024}									
M_{5-2024}									
C_{5-2024}									
M_{6-2024}									
C_{6-2024}									
M_{7-2024}					11.5 km to the Herja mine				
C_{7-2024}									
M_{8-2024}									
C_{8-2024}									
M_{9-2024}									
C_{9-2024}									
$M_{10-2024}$									
$C_{10-2024}$									
$M_{11-2024}$									
$C_{11-2024}$									
$M_{12-2024}$									
$C_{12-2024}$									
$M_{13-2024}$	A set of three grass samples were collected from	08-10 May	The sample collection location coincides with semi-	Ferneziu	5.5 km to the Herja mine	Maramureș Country/ Romania	2024	Rural	Near (~ 6/7 km distance) to the Herja Mine
$C_{13-2024}$									
$M_{14-2024}$									
$C_{14-2024}$									
$M_{15-2024}$									

C15-2024	each sampling point		permanent grazing ground utilized ty sheepfold						
M16-2024									
C16-2024									
M17-2024									
C17-2024									
M18-2024									
C18-2024									
M19-2024					7.5 km to the Herja mine				
C19-2024									
M20-2024									
C20-2024									
M21-2024									
C21-2024									
M22-2024									
C22-2024									
M23-2024									
C23-2024									
M24-2024									
C24-2024									
M25-2024	A set of three grass samples were collected from each sampling point	20-24 May	The sample collection location coincides with semi-permanent grazing ground utilized ty sheepfold	Firiza	16.5 km to settling pond Aurul	Maramureș Country/ Romania	2024	Rural	Near (~ 17 km distance) to settling pond mining (decant pond) Aurul from Tăuții de Sus
C25-2024									
M26-2024									
C26-2024									
M27-2024									
C27-2024									
M28-2024									
C28-2024									
M29-2024									
C29-2024									
M30-2024									
C30-2024									
M31-2024									
C31-2024									
M32-2024									
C32-2024									
M33-2024									
C33-2024									

M34-2024					17.0 km to settling pond Aurul				
C34-2024									
M35-2024									
C35-2024									
M36-2024									
C36-2024									
Background sheep's milks and cheese samples									
M37-2024	A set of three grass samples were collected from each sampling point	06-07 June	The sample collection location coincides with semi-permanent grazing ground utilized ty sheepfold	Tîrlișua	The control area was specifically chosen due to the absence of both detectable heavy metal contamination and high traffic volume.	Bistrița-Năsăud Country/ Romania	2024	Rural	The control area was meticulously selected to ensure minimal to no potential influence from heavy metal contamination from the outset.
C37-2024									
M38-2024									
C38-2024									
M39-2024									
C39-2024									
M40-2024									
C40-2024									
M41-2024									
C41-2024									
M42-2024									
C42-2024									
M43-2024									
C43-2024									
M44-2024									
C44-2024									
M45-2024									
C45-2024									
M46-2024									
C46-2024									
M47-2024									
C47-2024									
M48-2024									
C48-2024									

Table S5. Sampling site of sheep's serum from Ferneziu, Firiza (Maramureș area) and Tîrlișua (Bistrița-Năsăud), Romania.

Sample code	Number of samples	Harvest period	Soil details	Geographical origin	Distance from the source of pollution (~) km	Area/ Country	Year of harvest	Environment	Anthropogenic influence
Serum sheep's samples exposed to anthropogenic sources of heavy metals pollution									
S1-2024<br>S2-2024<br>S3-2024<br>S4-2024<br>S5-2024<br>S6-2024	A set of three sheep's serum samples were collected	08-10 May	The sample collection location coincides with semi-permanent	Ferneziu	8.0 km to the Herja mine	Maramureș Country/ Romania	2024	Rural	Near (~ 10/12 km distance) to the Herja Mine

S_{7-2024}<br>S_{8-2024}<br>S_{9-2024}<br>$S_{10-2024}$<br>$S_{11-2024}$<br>$S_{12-2024}$	from each sampling point		grazing ground utilized ty sheepfold		11.5 km to the Herja mine				
$S_{13-2024}$<br>$S_{14-2024}$<br>$S_{15-2024}$<br>$S_{16-2024}$<br>$S_{17-2024}$<br>$S_{18-2024}$<br>$S_{19-2024}$<br>$S_{20-2024}$<br>$S_{21-2024}$<br>$S_{22-2024}$<br>$S_{23-2024}$<br>$S_{24-2024}$	A set of three sheep's serum samples were collected from each sampling point	08-10 May	The sample collection location coincides with semi-permanent grazing ground utilized ty sheepfold	Ferneziu	5.5 km to the Herja mine<br>7.5 km to the Herja mine	Maramureș Country/ Romania	2024	Rural	Near (~ 6/7 km distance) to the Herja Mine
$S_{25-2024}$<br>$S_{26-2024}$<br>$S_{27-2024}$<br>$S_{28-2024}$<br>$S_{29-2024}$<br>$S_{30-2024}$<br>$S_{31-2024}$<br>$S_{32-2024}$<br>$S_{33-2024}$<br>$S_{34-2024}$<br>$S_{35-2024}$<br>$S_{36-2024}$	A set of three sheep's serum samples were collected from each sampling point	20-24 May	The sample collection location coincides with semi-permanent grazing ground utilized ty sheepfold	Firiza	16.5 km to settling pond Aurul<br>17.0 km to settling pond Aurul	Maramureș Country/ Romania	2024	Rural	Near (~ 17 km distance) to settling pond mining (decant pond) Aurul from Tăuții de Sus
Background sheep's serum samples									
$S_{37-2024}$<br>$S_{38-2024}$<br>$S_{39-2024}$<br>$S_{40-2024}$<br>$S_{41-2024}$<br>$S_{42-2024}$	A set of three sheep's serum samples were collected	06-07 June	The sample collection location coincides with semi-permanent	Tîrlișua	The control area was specifically chosen due to the absence of both	Bistrița-Năsăud Country/ Romania	2024	Rural	The control area was meticulously selected to ensure minimal to no potential influence from heavy metal contamination from the outset.

$S_{43-2024}$<br>$S_{44-2024}$<br>$S_{45-2024}$<br>$S_{46-2024}$<br>$S_{47-2024}$<br>$S_{48-2024}$	from each sampling point	grazing ground uti-lized ty sheepfold	detectable heavy metal contamination and high traf-fic volume.

Table S6. Details of the experimental animals

Area	Age of sheep	Health status	Color	Native sheep breed	Medications administered
Firiza	2-5	Good condition	White with black spots	Țurcană	Ivermectin-based anthelmintic treatment was conducted in the month of December
Ferneziu I	2-6	Good condition	A predominantly white coat with a black marking on the head	Țurcană	Anthelmintic treatment with Helmizol was administered on January 10, 2024
Ferneziu II	2-6	Good condition	White	Țurcană	Anthelmintic treatment with Evomec Plus was administered on January 2, 2024
Tîrlișua	2-7	Good condition	The sheep exhibited a white coat on the body and nose, with a black pigmentation around the eyes	Țurcană	Anthelmintic treatment with Fasciozones was administered on February 15, 2024

Table S7. The program of the microwave oven Milestone START D Microwave Digestion System

Step	Target Temp (°C)	Pressure Max. (psi)	Temperature Ramp (min.)	Hold Time (min.)	Power (%)
			Soil		
1.	200	800	10	5	100
2.	220	800	15	20	100
3.	35-40	800	-	45 min. cool-ing	-
			Green grass		
1.	85	800	4	5	100
2.	145	800	9	3	100
3.	200	800	4	3	100
4.	200	800	14	10	100
5.	35-40	800	-	45 min. cool-ing	-
			Sheep milk		
1.	145	800	10	5	100
2.	200	800	20	15	100
3.	35-40	800	-	45 min. cool-ing	-
			Sheep cheese		
1.	90	800	7	3	100
2.	170	800	5	3	100
3.	210	800	5	3	100
4.	201	800	20	10	100
5.	35-40	800	-	45 min. cool-ing	-
			Blood serum		
1.	85	800	2	3	100
2.	135	800	4	3	100
3.	230	800	5	5	100
4.	230	800	15	20	100
5.	35-40	800	-	45 min. cool-ing	-

Table S8. Instrumental (a) and data acquisition (b) parameters of ICP-MS

(a) Instrumental parameters		(b) Data acquisition parameters for quantitative mode	
RF power/W	1.4 kW	Measuring mode	Standard (Ar 5.0) Q Cell (Collision Cell) (He 6.0)
Argon (Ar) gas flow, Helium (He) gas flow		Point per peak	3
Nebulizer	1.0 L/min.	Scans/Replicate	7
Plasma gas low rate (Ar 5.0)	18.0 L/min.	Replicate/Sample	7
Auxiliary gas flow rate (He 6.0)	0.20 L/min.		
Lens voltage	37 V	Dwell time (ms)	3
Mirror lens right	32 V		
Mirror lens bottom	31 V		
Sample uptake rate	90 s	Integration time	1-5 ms

Temperature spray chamber	2.10 °C
Background correction	2 points/peak
Injector tube	quartz 2-mm id
Sample cone	Sample Cone 4450
Skimmer cone	Ni – Skimmer iCAP Q 0.5 mm insert version
Nebulizer	MicroMist Nebulizer 0.4 mL/min.

Table S9. Instrumental conditions for the determination of each element using ICP-MS technique.

Element	Correlation coefficient	LoD (μg/L)	LoQ (μg/L)	BEC (μg/L)
^{64}Cu	0.9997	0.035	0.139	0.236
^{65}Zn	0.9999	0.079	1.203	1.310
^{208}Pb	0.9996	0.151	0.231	0.649
^{111}Cd	0.9997	0.007	0.069	0.0031
^{60}Ni	0.9997	0.045	0.181	0.096
^{59}Co	0.9997	0.051	0.136	0.152
^{75}As	0.9999	0.006	0.743	0.018
^{52}Cr	0.9999	1.607	5.533	0.637
^{201}Hg	0.9999	0.043	0.137	0.128

LoD = Detection limit; LoQ = Quantification limit; BEC = Background equivalent concentration.

Table S10. Validation parameters of the analytical procedure for the determination of heavy metals (soil)

**Element**	**Certified reference material analysis**		**Validation parameters**	
	The result declared by de manufacture	The results obtained in our research	Recovery (%)	Uncertainty (%)
^{64}Cu (mg/kg)	34.6 ± 0.7	32.45 ± 2.78	96.7	19
^{65}Zn (mg/kg)	106 ± 3	100.1 ± 3.04	95.2	18
^{208}Pb (mg/kg)	18.9 ± 0.5	19.16 ± 0.84	96.0	11
^{111}Cd (mg/kg)	0.38 ± 0.01	0.37 ± 0.01	99.1	12
^{60}Ni (mg/kg)	88 ± 5	85.8 ± 2.36	85.3	16
^{59}Co (mg/kg)	13.4 ± 0.7	13.97 ± 0.75	95.5	21
^{75}As (mg/kg)	17.7 ± 0.8	16.7 ± 0.94	92.6	22
^{52}Cr (mg/kg)	103 ± 4	101.21 ± 2.36	98.8	18
^{201}Hg (mg/kg)	1.4 ± 0.08	1.29 ± 0.02	98.7	13

1. SRM – 2709a Standard Reference Material „San Joaquin Soil" Baseline Trace Element Concentration.

Table S11. Validation parameters of the analytical procedure for determination of heavy metals (green grass)

**Element**	**Certified reference material analysis**		**Validation parameters**	
	The result declared by de manufacture	The results obtained in own research	Recovery (%)	Uncertainty (%)
^{64}Cu (mg/kg)[a]	4.70 ± 0.14	4.42 ± 0.31	96.87	23
^{65}Zn (mg/kg)[a]	30.94 ± 0.55	31.84 ± 0.86	100.12	17
^{208}Pb (mg/kg)[b]	0.167 ± 0.015	0.164 ± 0.003	96.78	22
^{111}Cd (mg/kg)[a]	1.517 ± 0.027	1.687 ± 0.147	90.32	23
^{60}Ni (mg/kg)[a]	1.582 ± 0.041	1.784 ± 0.084	98.70	18
^{59}Co (mg/kg)[a]	0.5773 ± 0.071	0.6541 ± 0.321	107.18	24
^{75}As (mg/kg)[a]	0.1126 ± 0.024	0.1126 ± 0.062	117.89	16
^{52}Cr (mg/kg)[a]	1.988 ± 0.034	2.124 ± 0.070	92.69	21

^{201}Hg (mg/kg)[a]	0.0341 ± 0.0015	0.023 ± 0.017	95.84	10

[a] NIST – 1573a Tomato Leaves Standard Reference Materials; [b] NIST – 1575a Pine Needles (*Pinus taeda*) Standard Reference Materials.

Table S12. Validation parameters of the analytical procedure for determination of heavy metals (sheep milk and cheese)

Element	Certified reference material analysis		Validation parameters	
	The result declared by de manufacture	The results obtained in own research	Recovery (%)	Uncertainty (%)
^{64}Cu (mg/kg)[b]	1.09 ± 0.22	1.05 ± 0.03	94.78	22
^{65}Zn (mg/kg)[a]	151.07 ± 3.8	150.08 ± 0.11	99.12	17
^{208}Pb (mg/kg)[b]	0.0193 ± 0.004	0.0188 ± 0.026	97.84	24
^{111}Cd (mg/kg)[b]	0.0114 ± 0.0017	0.0118 ± 0.0048	95.36	21
^{60}Ni (mg/kg)[c]	0.056 ± 0.005	0.052 ± 0.003	92.78	16
^{59}Co (mg/kg)[b]	-	0.01656 ± 0.0054	105.87	21
^{75}As (mg/kg)[c]	0.0019 ± 0.001	0.0022 ± 0.006	112.89	19
^{52}Cr (mg/kg)[a]	1.045 ± 0.045	1.044 ± 0.014	99.67	20
^{201}Hg (mg/kg)[b]	0.060 ± 0.009	0.048 ± 0.009	99.45	15

[a] SRM – 1849a Infant/Adult Nutritional Formula I (milk-based); [b] ERM-BD150 – Skimmed Milk Powder (trace elements); [c] BCR-151 – Skim milk powder); SRM – 1549 Non-fat milk powder. .

Table S13. Validation parameters of the analytical procedure for the determination of heavy metals (Caprine Blood)

Element	Certified reference material analysis		Validation parameters	
	The result declared by de manufacture	The results obtained in our research	Recovery (%)	Uncertainty (%)
^{64}Cu (μg/L)[b]	1582 ± 96	1586 ± 102	102.78	21
^{65}Zn (μg/L)[b]	880 ± 24	856 ± 11	102.08	19
^{208}Pb (μg/dL)[a]	0.415 ± 0.0012	0.478 ± 0.0001	96.0	11
^{111}Cd (μg/L)[a]	0.0319 ± 0.0062	0.0317 ± 0.005	98.3	13
^{60}Ni (μg/L)[b]	0.99 ± 0.24	0.98 ± 0.05	91.5	21
^{59}Co (μg/L)[b]	1.22 ± 0.04	1.18 ± 0.06	99.6	22
^{75}As (μg/L)[a]	21.67 ± 0.15	23.04 ± 0.21	95.7	19
^{52}Cr (μg/L)[b]	0.33 ± 0.06	0.36 ± 0.03	101.2	22
^{201}Hg (μg/kg)[a]	0.017 ± 0.011	0.016 ± 0.01	98.2	13

[a] SRM – 955c Standard Reference Material Toxic Metals in Caprine Blood; [b] SRM – 1598a Standard Reference Material Inorganic Constituents in Animal Serum.

Figure S2. Comparing soil sample value ranges across sampling locations.

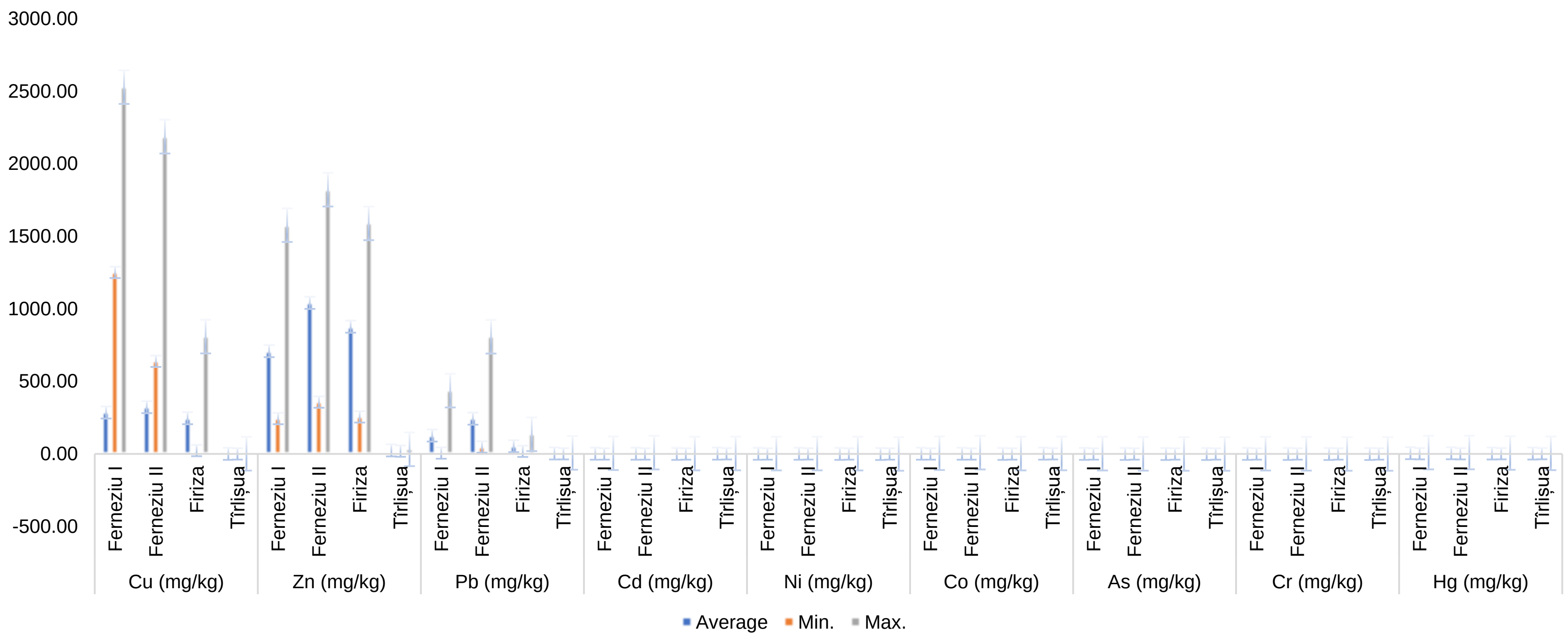

Figure S3. Soil sample value summary: mean, minimum, and maximum.

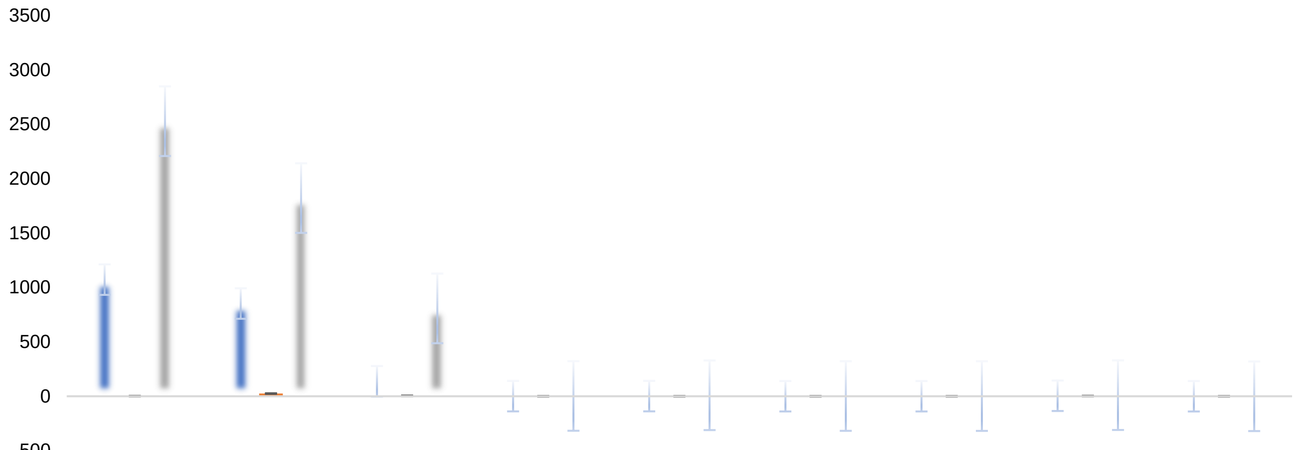

	Cu (mg/kg)	Zn (mg/kg)	Pb (mg/kg)	Cd (mg/kg)	Ni (mg/kg)	Co (mg/kg)	As (mg/kg)	Cr (mg/kg)	Hg mg/kg
Average	1072.35	852.78	137.73	1.14	1.44	0.36	0.35	4.58	0.00
Min.	0.89	24.66	3.75	0.00	0.00	0.00	0.00	1.57	0.00
Max.	2528.20	1821.40	807.59	2.94	9.02	2.13	1.73	10.01	0.00

Figure S4. Comparing green grass sample value ranges across sampling locations.

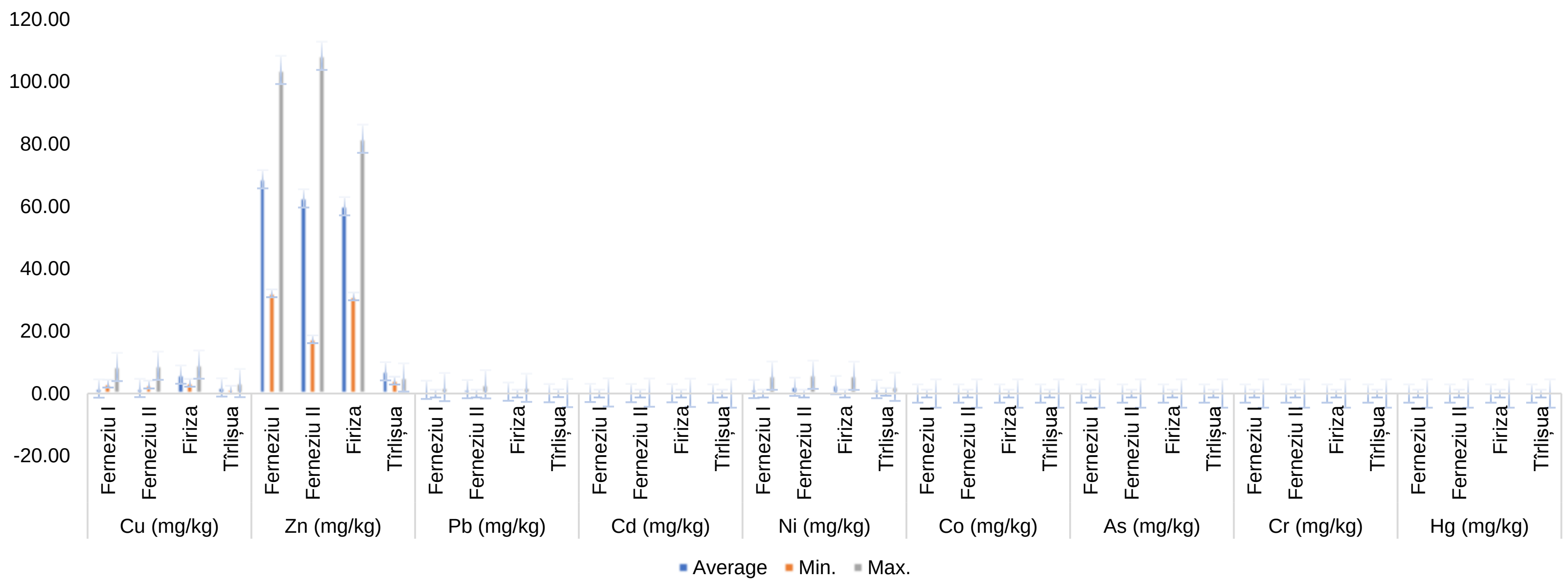

Figure S5. Green grass sample value summary: mean, minimum, and maximum.

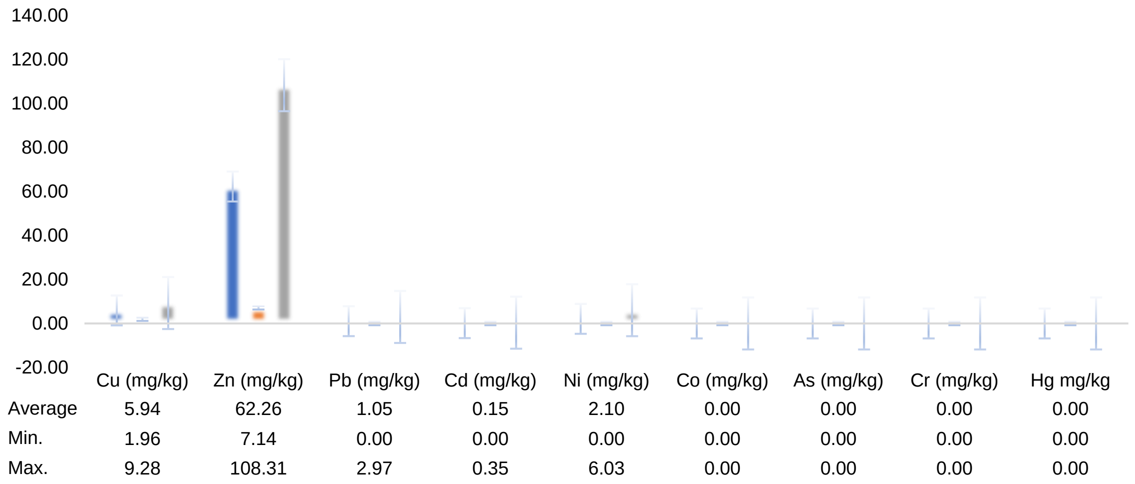

Figure S6. The mean, minimum, and maximum concentrations of elements of interest were determined for milk and sheep cheese samples. Only elements exceeding the detection limit were included in the analysis.

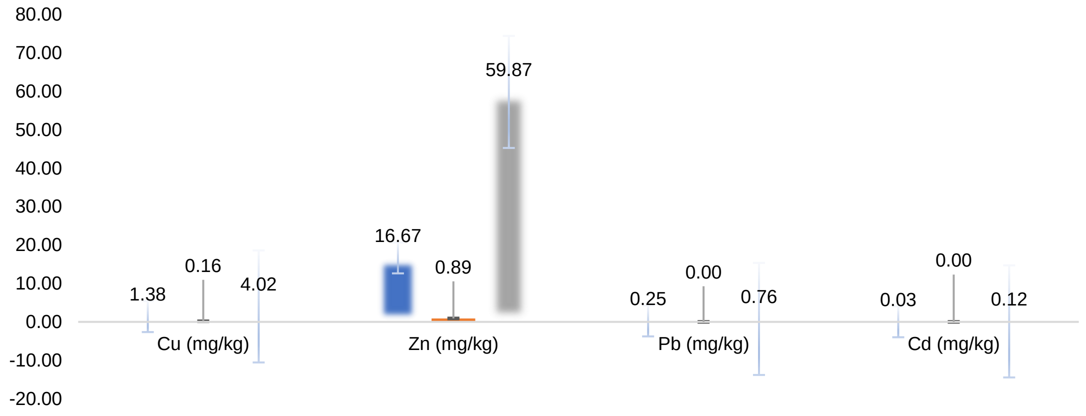

Figure S7. The mean, minimum, and maximum concentrations of elements of interest exceeding the detection limit were determined separately for milk and sheep cheese samples.

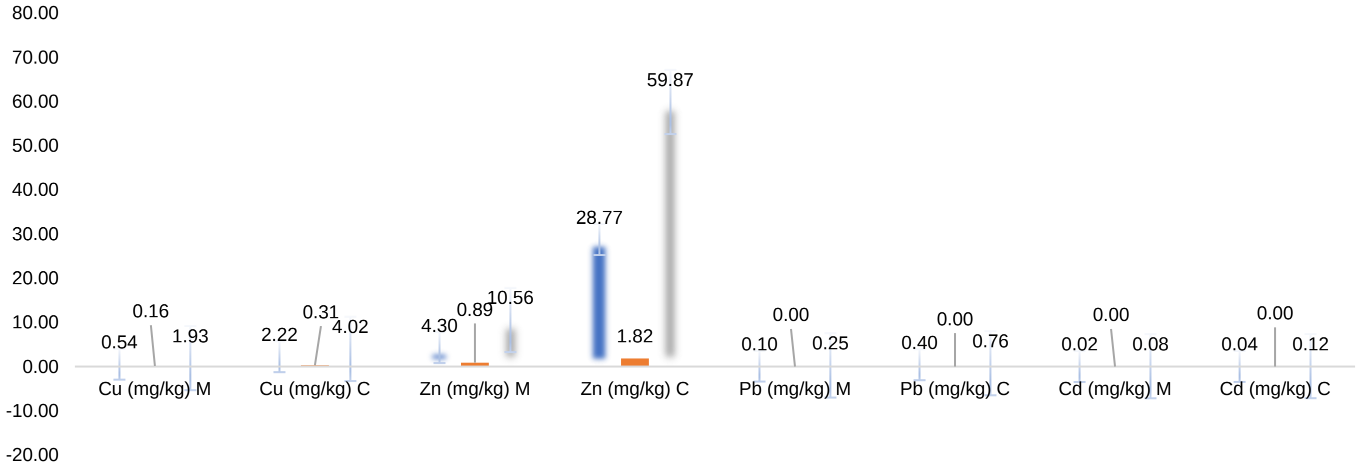

Figure S8. The mean, minimum, and maximum concentrations of elements of interest exceeding the detection limit were determined separately for serum sheep samples.

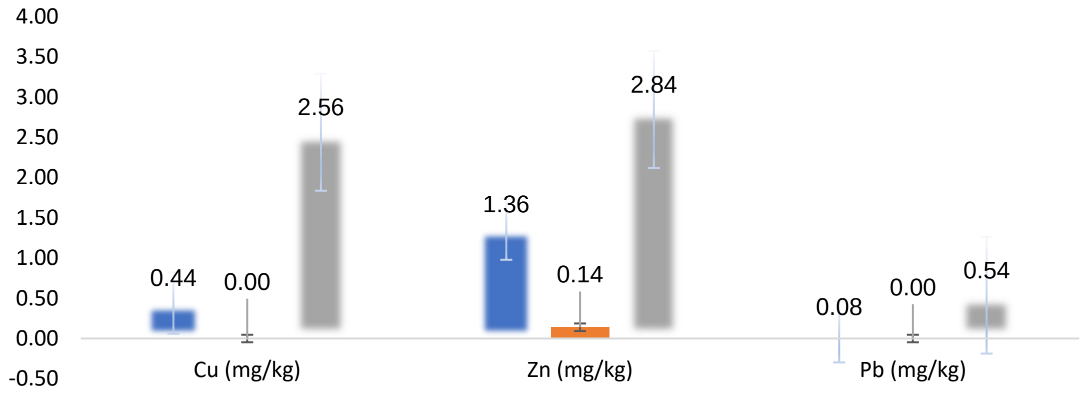

Supplement: Supplementary file 1 [file toxics-12-00752-s001.zip › toxics-3206439-supplementary.pdf]
